# Neuronal MAP kinase p38α inhibits c-Jun N-terminal kinase to modulate anxiety-related behaviour

**DOI:** 10.1038/s41598-018-32592-y

**Published:** 2018-09-24

**Authors:** Kristie Stefanoska, Josefine Bertz, Alexander M. Volkerling, Julia van der Hoven, Lars M. Ittner, Arne Ittner

**Affiliations:** 1 Dementia Research Unit, School of Medical Sciences, The University of New South Wales, Sydney, NSW 2052 Australia; 2 Dementia Research Centre, Faculty of Medicine and Health Sciences, Macquarie University, Sydney, NSW 2109 Australia

## Abstract

Modulation of behavioural responses by neuronal signalling pathways remains incompletely understood. Signalling via mitogen-activated protein (MAP) kinase cascades regulates multiple neuronal functions. Here, we show that neuronal p38α, a MAP kinase of the p38 kinase family, has a critical and specific role in modulating anxiety-related behaviour in mice. Neuron-specific *p38α*-knockout mice show increased levels of anxiety in behaviour tests, yet no other behavioural, cognitive or motor deficits. Using CRISPR-mediated deletion of *p38α* in cells, we show that p38α inhibits c-Jun N-terminal kinase (JNK) activity, a function that is specific to p38α over other p38 kinases. Consistently, brains of neuron-specific *p38α*-knockout mice show increased JNK activity. Inhibiting JNK using a specific blood-brain barrier-permeable inhibitor reduces JNK activity in brains of *p38α*-knockout mice to physiological levels and reverts anxiety behaviour. Thus, our results suggest that neuronal p38α negatively regulates JNK activity that is required for specific modulation of anxiety-related behaviour.

## Introduction

Mitogen-activated protein (MAP) kinases are centrally involved in signal transduction of mammalian cells. The MAP kinase families c-Jun N-terminal kinases (JNKs) and p38 MAP kinases – together also termed stress-activated protein kinases (SAPKs) – were mostly studied in the context of cellular stressors such as inflammatory cytokines and other danger signal molecules, UV radiation or osmotic stress^1^. In fact, p38 was discovered as a kinase responsive to inflammatory stimuli^2^. Four individual genes termed *p38α* (*MAPK14*), *p38β* (*MAPK11*), *p38γ* (*MAPK12*) and *p38δ* (*MAPK13*) encode p38 isoforms in mammalian organisms^3^. Despite significant similarity (~60% primary sequence identity), non-redundant functions in different cell types have been discovered for the individual *p38* genes, which suggest physiological functions of p38 kinases beyond stressor-related signalling^4–7^.

While substantial work has been done on p38 isoforms in non-neuronal cells (reviewed in^7–9^), non-redundant functions of p38 isoform in neurons and cognitive processes are incompletely understood. Due to the specificity of compound inhibitors towards p38α, studies have focussed on functions of this p38 kinase and have suggested roles for neuronal p38α in neurodegeneration^10–13^. Results from *p38α* gene-targeted mice imply a specific contribution of p38α in dorsal Raphe nucleus neurons to opioid-induced addictive behaviour^14,15^. Thus, insights on behavioural regulation by neuronal p38α have mainly been obtained from gene targeting in specific subsets of neurons. We recently showed that pan-neuronal *p38α* is dispensable for modulating progression of excitotoxic seizures induced by the *γ*-aminobutyric acid (GABA) antagonist pentylenetetrazole^5^. *p38α* may yet be involved in other modes of neurotoxicity^12,16^. However, effects of a pan-neuronal *p38α* deletion on behaviour are not known.

Genetic deletion of *p38α* has been reported for different cell types, such as in hepatocytes, keratinocytes or in cells of the immune system^17,18^. Deletion of *p38α* in early hematopoietic progenitor cells as well as in fibroblasts results in cell-autonomous hyperproliferation^19^. Interestingly, these models show increased JNK activity in the absence of *p38α*, suggesting that JNK activity is regulated by p38α^9^. Hepatocyte-specific *p38α* deletion sensitizes liver to cytokine signalling due to enhanced activity of the JNK pathway^17^. Furthermore, hepatocytes deficient in *p38α* show enhanced capacity for proliferation that is dependent on increased activity of the JNK pathway^19^. JNK inhibition can reduce levels of cellular proliferation in myoblasts after deletion of *p38α* suggesting that this cross-talk is of functional relevance^20^. However, whether cross-regulation of JNK MAPK signalling by p38α is present in neurons and whether it has implications for behavioural or cognitive functions in mammalian organisms is unknown.

Here, we show that pan-neuronal deletion of p38α in mice results in increased anxiety. We show that inhibition of the JNK pathway by p38α translates also to the brain. CRISPR-mediated targeting of *p38α* results in low levels of p38α expression and concomitantly uncontrolled activation of JNK. Furthermore, treatment with a central nervous system (CNS)-penetrant JNK- specific inhibitor reverts abnormally high levels of active JNK and anxiety levels in *p38α* knockout mice. Thus, our data suggest anxiety-related behaviour in mice is modulated through inhibition of JNK by neuronal p38α.

## Results and Discussion

### Neuronal *p38α* knockout results in altered anxiety-related response

Previous studies have reported the effects of conditional deletion of *p38α* in the CNS, specifically targeting dopaminergic^15^ or serotonergic neurons^21^. To address outcomes of pan-neuronal deficiency of p38α, we crossed *p38α* floxed (*p38α*^lox^) mice^22^ with mice that express cre recombinase under control of the pan-neuronal murine *Thy1.2* promoter^23^. Efficient neuron-restricted deletion in the resulting *p38α*^ΔNeu^ mice was confirmed by immunostaining of p38α and neuronal marker NeuN or astrocytic marker GFAP on hippocampal sections (Fig. S1A,B) and by immunoblot of cortical and hippocampal extracts (Fig. S1C). These results showed in addition that hippocampal levels of p38α protein are relatively higher as compared with cortical or cerebellar p38α levels (Fig. S1C). p38α expression was previously reported in CNS cell types other than neurons^14,24^. Consistently, immunoblots showed residual p38α signal in *p38α*^ΔNeu^ brain lysates (Fig. S1C). However, immunostaining did not show detectable p38α in *p38α*^ΔNeu^ mice likely due to sensitivity limitations. Pan-neuronal *p38α* knockout mice reproduced at Mendelian ratio, were phenotypically normal and showed normal body weight gain as well as metabolism (Fig. S1D,E).

**Figure 1:**
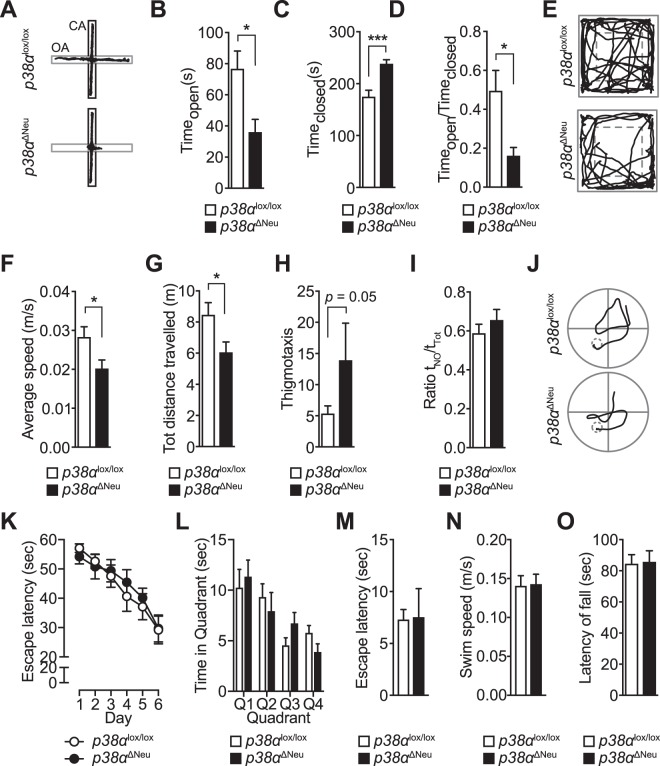
Mice with neuron-specific *p38α* deletion show abnormal anxiety-related responses. (**A**–**D**) Anxiety-related response in mice was tested for 5 minutes using the elevated plus maze (EPM) with *p38α*^lox/lox^ and *p38α*^ΔNeu^ mice. (n = 10–11) (**A**) Representative EPM traces. Traces reflect movement within the first minute of testing. OA, open arm; CA, closed arm. (**B**) Time in the open arms (**C**) Time in the closed arms. (**D**) Ratio of time in open/time in closed arms. (**E**–**H**) Activity in a novel environment was addressed using the open field paradigm (OFT) with *p38α*^lox/lox^ and *p38α*^ΔNeu^ mice. (n = 10–16) (**E**) Representative OFT traces. (**F**) Average speed in OFT (**G**) total distance covered in OFT. (**H**) Thigmotaxis index in OFT (**I**) Object recognition memory was addressed using the novel object recognition test (NOR) with *p38α*^lox/lox^ and *p38α*^ΔNeu^ mice. (n = 10–16) Ratio of time spent with novel object/time spent with familiar object is shown. (**J**–**N**) Spatio-temporal memory acquisition and retrieval was tested using the Morris water maze (MWM) with *p38α*^lox/lox^ and *p38α*^ΔNeu^ mice. (n = 8–10) (**J**) Representative MWM traces on acquisition day 6 (**K**) MWM acquisition curve on days 1–6 (**L**) MWM quadrant occupancy during probe trial on day 7. (**M**) Escape latency during visual cued trial on day 8 (**N**) Average swim speed during probe trial on day 7. (**O**) Motor assessment using the Rotarod with *p38α*^lox/lox^ and *p38α*^ΔNeu^ mice. (n = 13–14) Average latency to fall is shown. Values are mean ± S.E.M. (Student’s t-test) ^***^p < 0.001, ^*^p < 0.05 ns, non-significant.

To address functional consequences of neuronal deletion of *p38α* on behavioural and cognitive performance, we subjected 6-month old *p38α*^ΔNeu^ and *p38α*^lox/lox^ control mice to a series of behaviour tests. To test anxiety-related behaviour based on the natural aversion of mice for open and elevated areas, we subjected mice to the elevated plus maze (EPM) paradigm^25^. Strikingly, *p38α*^ΔNeu^ mice showed significantly lower occupancy of the open arms and higher occupancy of the closed arms than *p38α*^lox/lox^ controls during EPM testing (Fig. 1A–C), suggesting increased anxiety. Accordingly, the overall ratio of time spent in open to time spent in closed arms was approximately 3-fold lower in *p38α*^ΔNeu^ mice (Fig. 1D). Number of entries into arms and average speed were similar between both groups of mice, while there was a trend toward reduced time spent in the centre for *p38α*^ΔNeu^ mice (Fig. S2A–D). A tendency towards lower occupancy of open arms in the EPM was found in *p38α*^ΔNeu^ mice at 10–14 weeks of age, suggesting this phenotype develops with age (Fig. S2E–H). Taken together, these data suggest that neuronal *p38α* controls levels of anxiety in mice.

The open field paradigm tests locomotion, explorative and anxiety-related behaviour in a novel environment^5^. 6-month-old *p38α*^ΔNeu^ mice showed significantly lower levels of movement and total distances covered in the open field test as compared to *p38α*^lox/lox^ controls (Fig. 1E–G). Though it did not reach statistical significance (p = 0.05), *p38α*^ΔNeu^ mice showed a trend towards more thigmotaxis, i.e. movement restricted to the periphery of the open field arena (Fig. 1H). Younger mice (10–14 weeks of age) did not show significant differences in the open field paradigm (Fig. S2I,J). Both, reduced movement and increased tendency towards thigmotaxis indicate enhanced anxiety in mice in this test paradigm^26^. Thus, the open field testing suggests altered anxiety-related responses in mice lacking neuronal *p38α* at 6 months, in line with our findings from EPM testing at this age.

Next, we addressed memory function; We used the novel object recognition (NOR) paradigm to address recognition memory^27^. We found no significant differences between *p38α*^ΔNeu^ mice and *p38α*^lox/lox^ controls subjected to NOR testing, suggesting that depletion of neuronal *p38α* does not affect recognition memory (Figs 1I and S2E). To assess spatial learning and memory, we used the Morris Water Maze task (MWM)^5^. Both *p38α*^ΔNeu^ and *p38α*^lox/lox^ controls showed similar learning during the acquisition phase as indicated by escape path lengths on day 6 (Fig. 1J) and progressive reduction in escape latencies (Fig. 1K). Performance during the probe trials was similar in both *p38α*^ΔNeu^ and *p38α*^lox/lox^ mice, indicating that spatial memory was unaffected by lack of neuronal *p38α* (Fig. 1L). Escape latency and swim speed during the visual cued test phase were similar in both *p38α*^ΔNeu^ mice and *p38α*^lox/lox^ controls (Fig. 1M,N), suggesting comparable visual and motor performance under MWM test conditions. Overall, our results from NOR and MWM testing suggest normal learning and memory in *p38α*^ΔNeu^ mice.

Lastly, we addressed motor performance in *p38α*^ΔNeu^ mice and *p38α*^lox/lox^ controls using the accelerated rotating rod test (Rotarod)^5^ (Fig. 1O), the pole test^5,28^ (Fig. S2F), and by measuring grip strength^5^ (Fig. S2G). Performance of *p38α*^ΔNeu^ mice and *p38α*^lox/lox^ controls was indistinguishable in all motor tests, suggesting that neuronal *p38α* depletion does not alter motor coordination or muscle function. In summary, using a battery of behaviour, cognitive and motor tests we revealed a specific involvement of neuronal *p38α* in anxiety-related behaviour.

### Active *p38α* impairs activation of JNK in cultured cells

**Figure 2:**
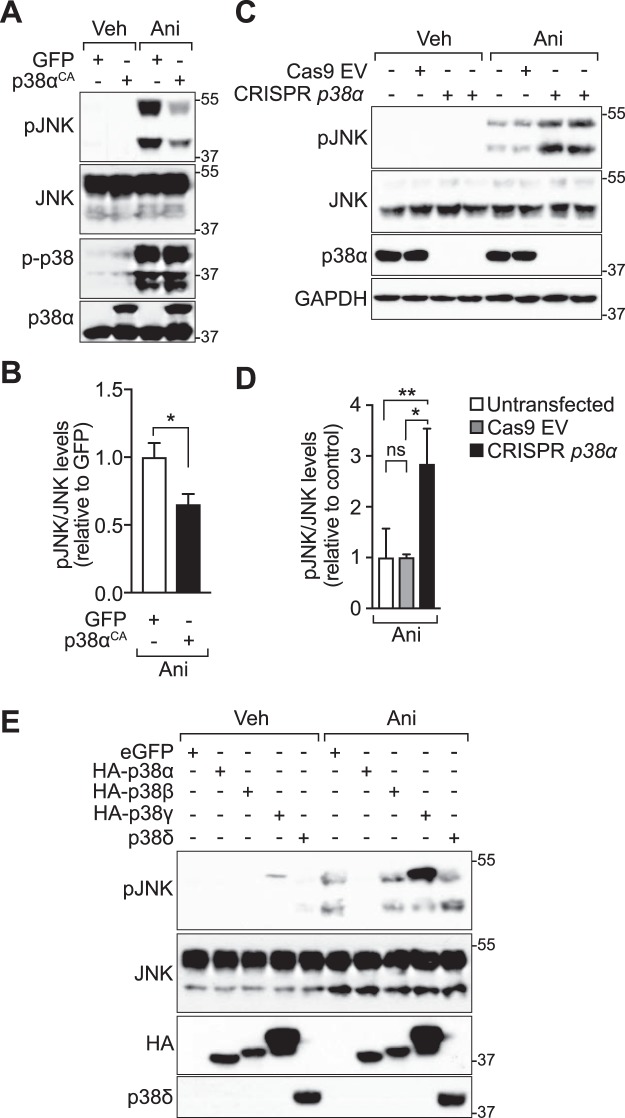
p38α impairs activation of JNK. (**A**) Expression of active p38α inhibits stimulated JNK activation in cultured cells. 293 T cells were transfected with constructs expressing either enhanced green fluorescent protein (eGFP) as control or constitutively active p38α (p38α^CA^). After stimulation with anisomycin (ANI; 25 ng/ml for 30 minutes) or vehicle (VEH), cell lysates were prepared for immunoblots for pThr183/pTyr185 JNK (pJNK), JNK (Cell Signaling Technologies), pThr180/Tyr182 p38 (p-p38) and p38α. GAPDH, loading control. (**B**) Quantification of immunoblots from three independent experiments (Student’s t-test) ^***^p < 0.001 (**C**) CRISPR/Cas9-mediated ablation of *p38α* increases stimulated JNK activation. eGFP-sorted 293 T cells transfected with Cas9 and *p38α* gRNA-expressing constructs (CRISPR *p38α*) or Cas9-expressing empty vector (Cas9 EV). Two different *p38α* gRNAs were co-transfected. After stimulation with anisomycin (ANI; 25 ng/ml for 30 minutes) or vehicle (VEH), cell lysates were prepared for immunoblots for pThr183/pTyr185 JNK (pJNK), JNK (Sigma) and p38α. GAPDH, loading control. (**D**) Quantification of immunoblots from three independent experiments (Student’s t-test) ^***^p < 0.001 (**F**) Of the four p38 MAPKs, only p38α inhibits JNK activation. 293 T cells were transfected with constructs expressing constitutively active hemagglutinin (HA)-tagged p38α (p38α^CA^), HA-tagged p38β (p38β^CA^), HA-tagged p38γ (p38γ^CA^) or p38δ (p38α^CA^). Cells expressing eGFP served as control. After stimulation with anisomycin (ANI; 25 ng/ml for 30 minutes) or vehicle (VEH), cell lysates were prepared for immunoblots for pThr183/pTyr185 JNK (pJNK), JNK, pThr180/Tyr182 p38 (p-p38), HA and p38δ. GAPDH, loading control. Representative blots from two experiments are shown.

Previous reports have shown increased activation of JNK in different cell types lacking *p38α*^17–19,29^, suggesting a regulation of JNK activity by p38α. This increased JNK activity has been shown to have diverse functional consequences in these cell types^17,19,30^. To extend these studies, we addressed whether p38α could affect JNK activity in a heterologous cell model. Therefore, we transiently transfected 293 T human embryonic kidney cells with constitutively active *p38α* (*p38α*^CA^) or GFP as a control. Using anisomycin, a potent stimulator of JNK activity, we addressed JNK activation in these cells using immunoblotting of cell lysates for phosphorylated JNK (p-JNK), a marker for activation of this kinase^31^. Anisomycin treatment (25 μg/ml, 30 minutes) induced JNK activation much less in p38α^CA^-expressing cells as compared to GFP-expressing controls (Fig. 2A,B). Activation of endogenous p38 was induced to a similar extent by anisomycin treatment in both experimental and control conditions (Fig. 2A).

We next addressed JNK activation when *p38α* expression was abolished. To achieve targeted disruption of the *MAPK14* gene in human 293 T cells, we employed CRISPR/Cas9 genome editing^32^. Excision of exon 1 in the murine *Mapk14* gene is sufficient to ablate gene product^22^. The murine and human *p38α* loci have similar exon structure, and exon 1 contains the start codon in both species (Fig. S3A). Therefore, we targeted exon 1 in the human *MAPK14* by CRISPR. A combination of two guide RNAs targeting the *MAPK14* gene was efficient in abolishing detectable levels of p38α (Figs 2C,D and S3B). This resulted in increased activation of JNK upon stimulation with anisomycin as compared with Cas9-expressing control cells (Fig. 2C,D). Thus, modulation of p38α inversely correlates with stimulus-dependent JNK activation in cultured cells.

We next addressed specificity among p38 MAP kinase family members for this effect on JNK activation. Expression of active p38β, p38γ or p38δ did not result in lower JNK activation levels after stimulation with anisomycin (Fig. 2E). Thus, an inhibitory effect of active p38 kinase on JNK activity appears specific to the p38α family member of p38 kinases. Taken together, our data confirm p38α-mediated inhibition of JNK activation as a cell-autonomous mechanism prevalent in different mammalian cell types and further suggest that this function is unique to p38α compared with the other p38 kinases.

### Neuronal *p38α* deletion results in increased JNK activation

**Figure 3:**
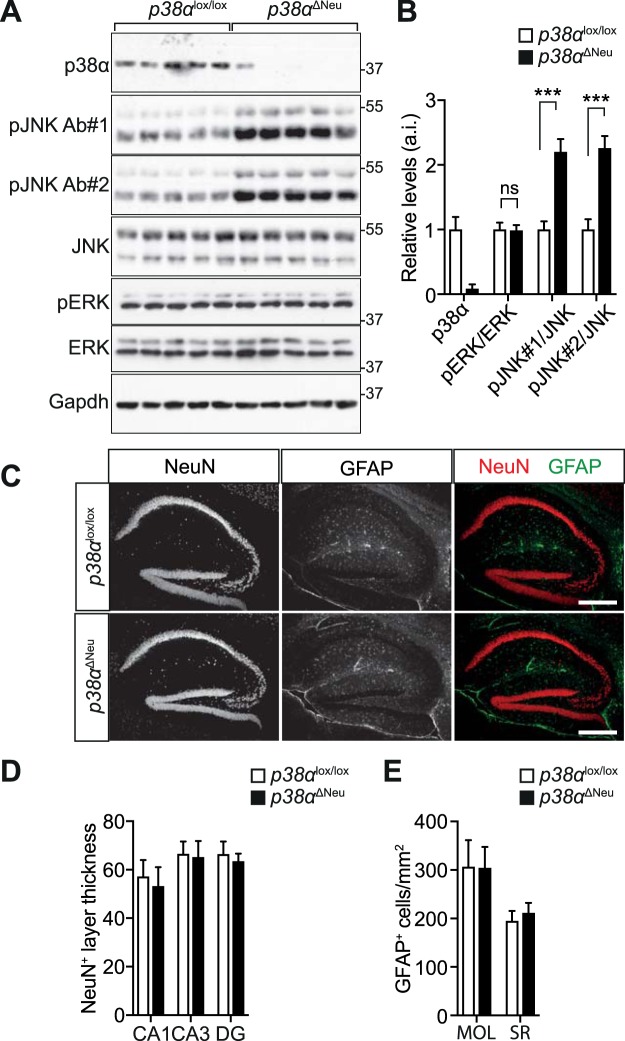
Neuron-specific *p38α* deletion results in increased hippocampal JNK activation in mice. (**A**) Immunoblots of hippocampal lysates (RIPA buffer with 10 µM okadaic acid) from *p38α*^lox/lox^ and *p38α*^ΔNeu^ mice (n = 5) were probed for pThr183/pTyr185 JNK (pJNK) using 2 independent antibodies (pJNK Ab#1 and Ab#2), JNK, pThr202/pTyr204 ERK (pERK), ERK and p38α. Gapdh, loading control. Note the high levels of pJNK in *p38α*^ΔNeu^ as compared to *p38α*^lox/lox^. (**B**) Quantification of immunoblots shown in (**A**). p38α levels are expressed relative to Gapdh levels. pJNK and pERK levels are expressed relative to total JNK and total ERK, respectively, and normalized to Gapdh levels. (n = 5) values are mean ± S.E.M. (Student’s t-test) ^***^p < 0.001; ns, non-significant. (**C**) Hippocampal sections (5 µm) of *p38α*^lox/lox^ and *p38α*^ΔNeu^ mice were prepared and immunostained for astrocytic marker GFAP and neuronal nuclear marker NeuN. Scale bar, 100 µm. Note the similar hippocampal structure, neuronal and astrocytic numbers in *p38α*^lox/lox^ and *p38α*^ΔNeu^ mice. (**D**) Thickness of NeuN^+^ cell layers in the hippocampus of *p38α*^lox/lox^ and *p38α*^ΔNeu^ mice. CA1, cornu ammonis 1; CA3, cornu ammonis 3; DG, dentate gyrus. (n = 5) values are mean ± S.E.M. (Student’s t-test) ns, non-significant. (**E**) Numbers of GFAP-positive astrocytic cells in the hippocampus of *p38α*^lox/lox^ and *p38α*^ΔNeu^ mice. MOL, molecular layer; SR, stratum radiatum. (n = 5) values are mean ± S.E.M. (Student’s t-test) ns, non-significant.

We next addressed whether neuronal deletion of p38α would result in higher JNK activity in the CNS. Addressing JNK activation first by immunoblotting, we found higher levels of pJNK in hippocampal lysates of *p38α*^ΔNeu^ mice as compared to *p38α*^lox/lox^ controls at 6 months of age and under physiological conditions (Fig. 3A,B). Furthermore, we did not find differences in levels of activated extracellular signal-regulated kinase (ERK) between *p38α*^ΔNeu^ and *p38α*^lox/lox^ control mice (Fig. 3A,B), suggesting that deficiency of p38α does not affect the ERK MAP kinase cascade. Deletion of *p38α* in hematopoietic lineage or in fibroblasts results in elevated proliferation and cell numbers^19^. To address whether neuronal deletion of *p38α* affects numbers of neurons, we sectioned brains from *p38α*^ΔNeu^ and *p38α*^lox/lox^ control mice at 6 months of age and performed Nissl staining or immunostaining for NeuN, a marker for neuronal nuclei^33^. Nissl staining showed normal development and size of all brain structures. Furthermore, we addressed thickness of NeuN-positive neuronal cell layers in the hippocampal formation (Figs 3C,D and S4). Cell layers were of similar thickness in CA1, CA3 and DG cell layers in both *p38α*^ΔNeu^ and *p38α*^lox/lox^ brains (Fig. 3C,D). This suggests that deletion of *p38α* has no significant effect on hippocampal development or on neuronal numbers in the hippocampus. GFAP immunoreactive cells, a marker of astrocytes in adult mice, were also similarly distributed in hippocampi of *p38α*^ΔNeu^ and *p38α*^lox/lox^ control mice (Fig. 3C,E). Cortical neuronal layer structure was similar between *p38α*^ΔNeu^ and *p38α*^lox/lox^ control mice. Increased JNK activation in neurons has been linked to cell death^34^. Despite higher levels of JNK activity in *p38α*^ΔNeu^ hippocampus, neuronal and astrocytic numbers appear normal, suggesting that elevated levels of active JNK are without adverse consequences on cell viability, development or glial reactivity in *p38α*^ΔNeu^ mice.

### JNK inhibitor reduces JNK activity in *p38α*-deficient mice to wild-type levels

**Figure 4:**
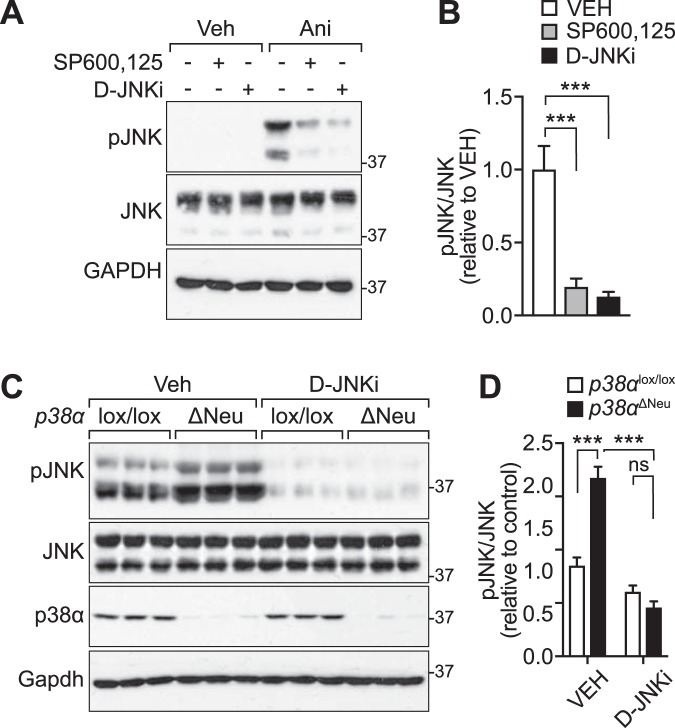
D-JNKi reduces JNK activation in neuronal *p38α* knockout mice to levels in control mice. (**A**) 293 T cells were treated with D-JNKi (1 μM), SP600125 (10 μM) for 30 minutes at 37 °C. After stimulation with anisomycin (ANI; 25 ng/ml for 30 minutes) or vehicle (VEH), cell lysates were prepared for immunoblots for pThr183/pTyr185 JNK (pJNK), JNK. GAPDH, loading control. (**B**) Quantification of immunoblots in (**A**). (n = 3) (Student’s t-test) ^***^p < 0.001. (**C**) Immunoblots of hippocampal lysates from *p38α*^lox/lox^ and *p38α*^ΔNeu^ mice 30 minutes post-application (i.p.) of D-JNKi (0.3 mg/kg body weight) or control vehicle (VEH). Immunoblots were probed for pThr183/pTyr185 JNK (pJNK), JNK, p38α and Gapdh. 30 minutes post injection of D-JNKi, activation levels of JNK were comparable between *p38α*^lox/lox^ and *p38α*^ΔNeu^ brains, whereas control-treated *p38α*^ΔNeu^ brains show consistently higher levels of active JNK than control-treated *p38α*^lox/lox^. (n = 5) (**D**) Quantification of immunoblots in (A). (n = 5) (Student’s t-test) ^***^p < 0.001.

We next addressed whether a mechanistic relationship existed between p38α-mediated inhibition of JNK activity and the anxiety-related behaviour of *p38α*-deficient mice. To modulate JNK activity, we compared reported properties of previously characterized pharmacological JNK inhibitors^35^. The D-amino acid peptide-based inhibitor D-JNKi was shown to impair JNK activity in rodents upon crossing the blood-brain-barrier (BBB) with high selectivity and a half-life that makes it suitable for acute *in vivo* studies of behavioural processes^36–40^. Cells treated with D-JNKi reduced JNK activation upon treatment with anisomycin (Fig. 4A), similar to SP600,125, as widely used JNK inhibitor^35^. This confirmed inhibition of JNK by D-JNKi.

Next, we injected *p38α*^*ΔNeu*^ and *p38α*^*lox/lox*^ controls with D-JNKi (0.3 mg/kg body weight i.p.^38^) or a vehicle control solution and addressed JNK activation levels in the hippocampus 30 minutes post-injection by immunoblotting. Hippocampal lysates of *p38α*^*ΔNeu*^ and *p38α*^*lox/lox*^ control mice that were injected with D-JNKi showed significantly lower levels of p-JNK as compared with lysates from vehicle treated mice (Fig. 4B,C). Notably, D-JNKi-injected *p38α*^*ΔNeu*^ mice showed levels of p-JNK that were comparable to vehicle-injected *p38α*^*lox/lox*^ control mice (Fig. 4B). These results suggest that increased JNK activation in *p38α*^*ΔNeu*^ mice is amenable to acute modulation by D-JNKi treatment.

### Inhibition of JNK restores anxiety-related behaviour in *p38α*^*ΔNeu*^ mice

**Figure 5:**
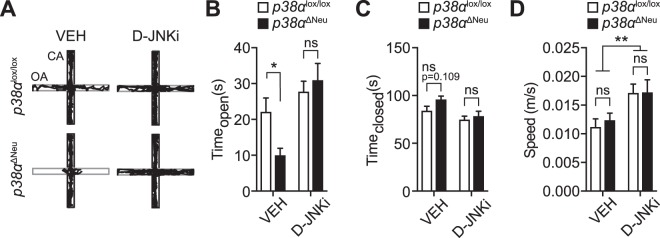
Inhibition of JNK restores anxiety-related behaviour in neuronal *p38α* knockout mice. (**A**–**D**) EPM tests (2 minutes) in *p38α*^lox/lox^ and *p38α*^ΔNeu^ mice 30 minutes post-application (i.p.) of D-JNKi (0.3 mg/kg body weight) or control vehicle (VEH). (n = 15–16). (**A**) Representative EPM traces reflect movement within the first 2 minutes of testing. OA, open arm; CA, closed arm. (**B**) Time in the open arms. (C) Time in the closed arms. (**D**) Ratio of time in open/time in closed arms. Values are mean ± S.E.M. (ANOVA) ^**^p < 0.01, ^*^p < 0.05 ns, non-significant.

We addressed functional consequences of acute reduction of JNK activation in *p38α*^*ΔNeu*^ mice on anxiety-related behaviour in the EPM paradigm. *p38α*^*ΔNeu*^ and *p38α*^*lox/lox*^ control mice were treated with D-JNKi (0.3 mg/kg body weight i.p.) or vehicle 30 minutes prior to EPM testing. As expected, vehicle-injected mice lacking neuronal *p38α* showed significantly lower occupancy of the open maze arms than *p38α*^*lox/lox*^ control mice after vehicle injection (Fig. 5A–C). However, both *p38α*^*ΔNeu*^ and *p38α*^*lox/lox*^ control mice spent similar amounts of time in open arms after injection with D-JNKi (Fig. 5A–C). The average speed of vehicle-treated *p38α*^*ΔNeu*^ and *p38α*^*lox/lox*^ control mice was comparable, as was the average speed of D-JNKi-treated *p38α*^*ΔNeu*^ and *p38α*^*lox/lox*^ control mice (Fig. 5D). However, D-JNKi application enhanced general locomotion in mice (Fig. 5D). These results suggest that lowering JNK activity using D-JNKi can acutely revert the altered anxiety-related behaviour in *p38α*^*ΔNeu*^ mice. This supports the mechanistic concept that neuronal p38α controls behavioural states relevant to anxiety through inhibition of JNK in the CNS. Thus, the p38α-JNK pathway can support physiological functions in the CNS related to anxiety behaviour.

Our results show that inhibition of JNK activation by neuronal p38α controls anxiety-related behaviour in mice. JNK activity is constitutively higher in mammalian brain as compared with other tissues^41^, suggesting that the brain maintains high JNK activity for physiological functions. JNK signalling has been previously linked to regulation of anxiety in rodents. *Jnk1*-deficient mice or mice after long-term treatment with D-JNKi present with reduced responses in anxiety-related behaviour^42,43^. Though other mouse models with specifically increased JNK activity in the CNS have not been reported, phenotypes of mice with genetic *mkp-1* deletion are of note; *mkp-1*-deficient mice show increased activity of JNK, ERK and p38 MAP kinase in the brain, are more resilient to stress and show decreased anxiety levels in the EPM^44^. However, the individual contributions of the different MAP kinase families to behaviour in *mkp-1* knockout mice remain unclear, leaving it uncertain whether the effects in *mkp-1*-deficient mice can be attributed to activity of JNK. Our results using JNK inhibitor D-JNKi in *p38α*^*ΔNeu*^ mice support the idea, however, that neuronal JNK activity specifically contributes to dysregulation of anxiety responses with increased anxiety behaviour in the EPM and open field paradigms. Though our experimental control is consistent with other studies using D-JNKi, further experiments using a scrambled D-amino acid peptide control may be needed in future studies, based on experience with similar peptide kinase inhibitors^45^.

During development, JNK1 is required for migration of cortical interneurons^46^. However, cortex and hippocampal formation of *p38α*^*ΔNeu*^ mice were histologically normal. Furthermore, the *Thy1.2* promoter-driven deleter strain we employed expresses cre recombinase postnatally in forebrain neurons^23^. The observed anxiety-related phenotype of *p38α*^*ΔNeu*^ mice during testing is unlikely due to developmental alterations, since JNK inhibition fully reverted the deficits. In addition, *p38α*^*ΔNeu*^ mice do not show overt neurological phenotypes and develop normally.

Aberrant protein kinase-mediated signal transduction in neurons may contribute to development and persistence of anxiety-related disorders such as depression^47^ and other psychiatric disorders^34^. Understanding molecular pathways that are important in these disorders is critical to inform development of anxiolytic drugs. Studies in mouse models suggest that p38 and JNK MAP kinases are both valuable targets in anxiety disorders^21,42^. Beyond anxiety-related disorders, inhibition of p38α has been suggested as a treatment for neurological diseases, including neurodegeneration^13,48–50^ and neuropathic pain^51^. Our study suggests that inhibition of neuronal p38α may result in altered anxiety-related behaviour, which is important to bear in mind when considering p38α inhibitors as a therapeutic option.

## Methods

### Mice

Mice with targeted (floxed) *mapk14* (*p38alpha*) allele on a C57BL6 background have previously been described^22^. Neuron-specific cre deleter strain Tg(Thy1-cre)^1Vln/J^ on a C57BL6 background has been described before^23^. Mice were housed in 12 hour/12 hour light-dark cycle with food ad libitum (Rodent chow; Gordon Specialty Feeds). Genotyping was performed by PCR on DNA isolated from tail biopsies using the following oligonucleotide primers: *p38α*^lox^fwd 5′TCCTACGAGCGTCGGCAAGGTG’3, *p38α*^lox^rev 5′AGTCCCCGAGAGTTCCTGCCTC3′, *Thy1.2*-Cre-fwd 5′GCGGTCTGGCAGTAAAAACTATC3′, *Thy1.2*-Cre-rev 5′GTGAAACAGCATTGCTGTCACTT3′. Experiments were performed with age-matched mice and equal distribution of genders in experimental groups. All animal experiments were approved by the Animal Care and Ethics Committee of the University of New South Wales. All animal experiments were performed in accordance with relevant guidelines and regulations.

#### Behaviour testing

Elevated plus maze: Anxiety-related behaviour was addressed in the elevated plus maze as previously described^25^. Briefly, mice were placed individually in an elevated 50 × 50 cm^2^ cross-shaped maze with 2 closed and 2 open arms (Stölting) in a brightly lit environment and movements were recorded. Starting position was in the centre of the maze. Mice had not been exposed to the EPM before. Maze was wiped with 70% ethanol between recordings. Movements were tracked using the AnyMaze software (Stölting).

Open field paradigm: Novelty-induced locomotion and anxiety-related behavior was assessed in the open field test paradigm as previously described^52^. Briefly, mice were placed individually in 40 × 40 cm^2^ boxes in dimly lit sound-insulated enclosures and movements were recorded for 15 minutes. Mice had not been exposed to open field paradigm before. Boxes were wiped with 70% ethanol between recordings. Tracking analysis (AnyMaze, Stölting) was either accumulated over entire recording period or split in 1-minute bins. Thigmotaxis index was calculated as ratio of time spent in the periphery of the OFT arena and the time spent in the centre of the OFT arena.

Morris water maze: Spatial learning/memory was tested in the Morris Water maze paradigm^5,53^. Briefly, a custom-built water tank for mouse Morris Water maze (122 cm diameter, 50 cm height) with white non-reflective interior surface in a room with low-light indirect lighting was filled with water (19–22 °C) containing diluted non-irritant white dye. Four different distal cues were placed surrounding the tank at perpendicular positions of the 4 quadrants. In the target quadrant (Q1), a platform (10 cm^2^) was submerged 1 cm below the water surface. Videos were recorded on CCD camera and analyzed using AnyMaze Software. For spatial acquisition, four trials of each 60 seconds were performed per session. The starting position was randomized along the outer edge of the start quadrant for all trials. To test reference memory, probe trials without platform were performed for a trial duration of 60 seconds, and recordings were analyzed for time spent within each quadrant. For visually-cued control acquisition (to exclude vision impairments), a marker was affixed on top of the platform and four trials (60 s) per session were performed. All mice were age and gender-matched and tested at 4 months of age. Mice that displayed continuous floating behavior were excluded. Genotypes were blinded to staff recording trials and analyzing video tracks. Tracking of swim paths was done using the AnyMaze software (Stolting). Average swimming speed was determined to exclude motor impairments.

Novel object recognition task: Object recognition was tested as previously described^27^. After a habituation phase of 15 minutes in the test arena consisting of a 40 × 40 cm^2^ box in a dimly lit sound-insulated enclosure, mice were faced with 2 identical objects for 10 minutes. After one hour, mice were faced with one familiar and a novel object in the same location for 5 minutes. Movements were videorecorded and analysed by tracking software (AnyMaze, Stolting). Time of interaction with objects were summed up and ratio of interaction with novel object and total object time was calculated.

Rotarod: Motor performance was tested on a 5-wheel Rota-Rod treadmill (Ugo Basile) in acceleration mode (5–60 rpm) over 120 (aged) or 180 (young) seconds^5^. The longest time each mouse remained on the turning wheel out of 3 attempts per session was recorded.

Grip strength: Grip strength was determined as previously described^5^. Briefly, the force required to pull mice off a metal wire was measured using a grip strength meter (Chatillon, AMETEK). Mice were placed such that they had a double grip on a thin metal wire attached to the meter, and they were pulled away from the meter in a horizontal direction until they let go, and a peak force (N) was recorded at the moment when the mice let go. The highest force from three attempts was recorded.

### Glucose metabolism

Glucose tolerance tests were done as previously reported^54^. After overnight fasting, mice were injected intraperitoneally (i.p.) with D-glucose (2 mg/g) and blood was sampled from tail vein at indicated time points. Glucose was measured on handheld glucometer (Abbott).

### Cell culture

293 T cells were cultured in complete growth medium consisting of Dulbecco’s Modified Eagle Medium (DMEM, Gibco), 10% FCS (Gibco), L-Glutamine, penicillin/streptomycin. 293 T cells were transfected by calcium precipitation or polyehtyleneimine (PEI) lipofection. Anisomycin (Sigma-Aldrich) was dissolved in dimethylsulfoxide and further diluted to final concentration in complete growth medium.

### CRISPR/Cas9 genome editing of the *MAPK14* locus

Design of guide RNAs (gRNAs) was performed as previously described^32^ using the Massachusetts Institute of Technology webtool (http://crispr.mit.edu/). gRNAs templates for *MAPK14* were: gRNA#1 5′-GAGGCCCACGTTCTACCGGC-3′ gRNA#2 5′-CTGCCGCTGGAAAATGTCTC-3′. gRNA template oligos were inserted into pX458 (Addgene; 48138) by oligocloning. 293 T cells were transfected using polyethyleneimine (PEI)^55^. 16 hours post-transfection, cells were trypsinized, washed with PBS and resuspended in FACS sorting buffer (2%FCS, 2 mM EDTA, PBS pH 7.4). eGFP-positive cells were sorted on a FACSAria (BD). Empty pX458-transfected cells were sorted as control culture. After sorting, cells were cultured for subsequent experiments and genomic DNA was isolated by isopropanol precipitation to address genome targeting of the *MAPK14* locus by PCR. Primers for *MAPK14* genotyping of 293 T cells were: forward: 5′-AGCGCAAGGTCCCCGCCCGGCTG-3′ reverse: 5′-ACCCTGCCCACAGCGGCCCCAGG-3′.

### p38 expression constructs

Plasmid constructs for expression of p38 isoforms were described previously^5^. Constitutively active variants were based on single amino acid exchange variants previously described^56^.

### Tissue lysates

Brain tissue was homogenised in RIPA buffer (50 mM Tris pH 8.0, 150 mM sodium chloride, 5 mM sodium ethylenediaminetetraacetate, 1 mM sodium vanadate, 1 mM sodium pyrophosphate, 20 mM sodium fluoride, 0.5% sodium deoxycholate, 0.1% sodium dodecyl sulfate, 1% nonident P40 and 0.1% protease inhibitor (Roche Applied Science, Sydney, Australia)) using a dounce homogenizer (Heidolph). Okadaic acid (10 μM) was added to RIPA buffer where indicated in the figure legend. Insoluble material was pelleted by centrifugation (16,000 g, 10 minutes, 4 °C). Supernatant was transferred to a new tube and protein concentration was determined by BCA assay (BioRad).

### Histology and immunofluorescence

Mice were transcardially perfused with phosphate-buffered saline (PBS pH 7.4) followed by 4% paraformaldehyde (PFA). Tissue was extracted and post-fixed in 4% PFA overnight. Tissue was processed in an Excelsior tissue processor (Thermo) for paraffin embedding. For frozen section, mice were transcardially perfused with 4% paraformaldehyde (PFA) in PBS pH 7.4, brain tissue was extracted and post-fixed in 4% PFA overnight. Tissue was then processed at 4 °C in 10% sucrose in PBS pH7.4 for 1 hour, in 20% sucrose in PBS pH 7.4 for 1 hour, followed by 30% sucrose PBS pH 7.4 overnight. Sections (10 μm) were prepared on a cryostat (Leica). Immunofluorescence staining was done as previously described^57^. Briefly, tissue sections (5 μm) were rehydrated, washed with phosphate buffered saline (PBS pH 7.4), permeabilised with 0.02% NP-40 in PBS and blocked with blocking buffer (3% horse serum/1% bovine albumin in PBS pH 7.4). Primary antibodies diluted in blocking buffer were incubated over-night at 4 °C or for 1 hour at room temperature. After washing with PBS, secondary antibodies diluted in blocking buffer with or without addition of DAPI to visualize cell nuclei were incubated for 1 hour at room temperature. Cells were then washed and mounted using anti-fade mounting medium (Prolong Gold, Life Technologies). Secondary antibodies used were coupled to Alexa 488, 555, 568 or 647 dyes (Molecular Probes). Epifluorescence imaging was done on a BX51 bright field/epifluorescence microscope (UPlanFL N lenses [∞/0.17/FN26.5]: 10×/0.3, 20×/0.5, 40×/0.75, 60×/1.25 oil and 100×/1.3 oil) equipped with a DP70 color camera (Olympus) using CellSens software (Olympus). Mean cell counts for GFAP^+^ cells in the molecular outer layer and stratum radiatum were normalized to cell density and expressed as number of GFAP^+^ cells per mm^2^ using a Java-based image processing program (ImageJ). For NeuN^+^ immunostaining, mean counts across layers CA1, CA3 and DG were normalized to the length and size of the respective areas and expressed as a measure of NeuN^+^ layer thickness.

### Immunoblotting

Immunoblotting was performed as previously described^4^. Signal was visualized by chemiluminescence on X-ray films or by digital acquisition on a ChemiDoc MP (Biorad). Densitometric quantification of Western blot results was performed using ImageJ 2.0.0-rc-49/1.51d (NIH). Antibodies used were phospho-T202/Y204 ERK (D13.14.4E; Cell Signaling Technologies), ERK (Cell Signaling Technologies), phospho-T183/Y185 JNK (81E11; Cell Signaling Technologies), phospho-T183/Y185 JNK (98F2; Cell Signaling Technologies), JNK1/2/3 (Cell Signaling Technologies), JNK (Sigma), phospho-T180/Y182 p38 (D3F9; Cell Signaling Technologies), p38α (Cell Signaling Technologies), GAPDH (Millipore), NeuN (Abcam), GFAP (Abcam).

### Statistical analysis

Statistical analysis was done using Graphpad Prism (v7.0c). For data comparisons of 2 experimental groups of data unpaired, two-tailed Student t-test was used. For comparisons for >2 groups of data, ANOVA was used. Data are expressed as mean ± S.E.M. unless stated otherwise in the figure legend.

## Electronic supplementary material

Supplementary information

## Data Availability

The datasets generated during and/or analysed during the current study are available from the corresponding author on reasonable request.
