# Supplementary material for: Neuronal MAP kinase p38α inhibits c-Jun N-terminal kinase to modulate anxiety-related behaviour
Source: Sci Rep. 2018 Sep 24;8:14296. doi: 10.1038/s41598-018-32592-y (PMC6155170; doi:10.1038/s41598-018-32592-y)

Supplementary information

Neuronal MAP kinase p38α inhibits c-Jun N-terminal kinase to modulate anxiety-related behaviour

Author(s)

Kristie Stefanoska[1*], Josefine Bertz[1*], Alexander M Volkerling[1], Julia van der Hoven[1], Lars M Ittner[1] & Arne Ittner[1]

Complete name(s) of institution(s)

[1]Dementia Research Unit, School of Medical Sciences, The University of New South Wales, Sydney, NSW 2052, Australia.

*These authors contributed equally to this work.

Correspondence should be addressed to: A. I. (Email: a.ittner@unsw.edu.au)

Supplementary Figure legends

Figure S1. Efficient deletion of *p38α*, normal body weight development and glucose tolerance in *p38α*$^{\Delta Neu}$ mice.

(**A**) Efficient neuron-restricted deletion of *p38α* in *p38α*$^{\Delta Neu}$ brains. Sections (10 μm) from brains of *p38α*$^{lox/lox}$ and *p38α*$^{\Delta Neu}$ mice were prepared and immunostained for p38α and neuronal nuclear marker NeuN. DAPI, nuclear counter staining. (n= 3-4) Scale bar, 25 μm. Note the absence of signal for p38α in neuronal cells. (**B**) Sections (10 μm) from brains of *p38α*$^{lox/lox}$ and *p38α*$^{\Delta Neu}$ mice were prepared and immunostained for p38α and astrocytic marker GFAP. (n= 2-4) Scale bar, 25 μm. (**C**) Deletion analysis of p38α protein in hippocampus, cortex, cerebellum and spinal cord. Immunoblots of hippocampal, cortical, cerebellar and spinal cord lysates from *p38α*$^{lox/lox}$ and *p38α*$^{\Delta Neu}$ mice (n=2-3) were probed for p38α. Gapdh served as loading control. Note the relatively higher levels of hippocampal p38α. (**D**) Bodyweight of male (*left*) and female (*right*) *p38α*$^{lox/lox}$ and *p38α*$^{\Delta Neu}$ mice. Body weight of *p38α*$^{lox/lox}$ and *p38α*$^{\Delta Neu}$ mice develop similarly in both genders. (n = 9-13) values are mean ± S.E.M. (multiple t-tests) ns, non-significant (**E**) Glucose tolerance tests in male *p38α*$^{lox/lox}$ and *p38α*$^{\Delta Neu}$ mice. Glucose tolerance is comparable between *p38α*$^{lox/lox}$ and *p38α*$^{\Delta Neu}$ mice. (n = 6) values are mean ± S.E.M. (2-way ANOVA) ns, non-significant

Figure S2. Additional information on behaviour tests with *p38α*$^{lox/lox}$ and *p38α*$^{\Delta Neu}$ mice.

(**A**) EPM time in the maze centre. (n = 10-11) Values are mean ± S.E.M. (Student's t-test) ns, non-significant (**B**) Number of entries into closed arms of EPM. (n = 10-11) Values are mean ± S.E.M. (Student's t-test) ns, non-significant (**C**) Number of entries into open arms of EPM. (n = 10-11) Values are mean ± S.E.M. (Student's t-test) ns, non-significant (**D**) Average speed in EPM. (n = 10-11) Values are mean ± S.E.M. (Student's t-test) ns, non-significant (**E-H**) EPM with 10-14-week-old *p38α*$^{lox/lox}$ and *p38α*$^{\Delta Neu}$ mice. (**E**) EPM time in the open arms. (n = 7-9) Values are mean ± S.E.M. (Student's t-test) ns, non-significant (**F**) EPM time in the closed arms. (n = 7-9) Values are mean ± S.E.M. (Student's t-test) ns, non-significant (**G**) EPM time in the maze centre. (n = 7-9) Values are mean ± S.E.M. (Student's t-test) ns, non-significant (**H**) EPM time in the open arms plotted for each 30-second interval. (n = 7-9) Values are mean ± S.E.M. (2-way ANOVA) ns, non-significant (**I-J**) Activity of 10-14-week-old *p38α*$^{lox/lox}$ and *p38α*$^{\Delta Neu}$ mice in the open field paradigm (OFT). (**I**) Total distance covered (n = 7-9) Values are mean ± S.E.M. (Student's t-test) ns, non-significant (**J**) Thigmotaxis index (n = 7-9) Values are mean ± S.E.M. (Student's t-

test) ns, non-significant (**K**) Total time spent with objects in the NOR test (n = 10-16) values are mean ± S.E.M. (Student's t-test) *** $p < 0.001$ * $p < 0.05$ ns, non-significant (**L**) Additional motor coordination testing: pole test with *p38α*$^{lox/lox}$ and *p38α*$^{\Delta Neu}$ mice. (n = 18-21) Values are mean ± S.E.M. (Student's t-test) ns, non-significant (**M**) Muscle strength was tested using the grip strength test with *p38α*$^{lox/lox}$ and *p38α*$^{\Delta Neu}$ mice. (n = 14-18) Values are mean ± S.E.M. (Student's t-test) ns, non-significant

Figure S3. Comparison of exonic structure of *p38α* genes in human and mouse

(**A**) Genomic alignment and comparison of exons 1-3 of human *MAPK14* and mouse *mapk14* genetic structure (Ensembl/Havana merged protein-encoding exon annotation). Exons 1-3 show similar distribution, length and position of the start codon of *p38α*.

(**B**) CRISPR/Cas9-mediated ablation of *p38α*. Schematic showing position of the 2 guide RNAs (*red arrows*) used for gene editing of exon 1 of the human MAPK14 gene. F, R, forward and reverse primers (*black bars*) used for PCR analysis of the locus yielding a 264-base pair (bp) amplicon in the wildtype locus. Genomic DNA from eGFP-sorted 293T cells transfected with Cas9 and *p38α* gRNA-expressing constructs (CRISPR *p38α*) or Cas9-expressing empty vector (Cas9 EV) was used as template for PCR. Note the band at ~260 bp in Cs9 EV control, which is absent in CRISPR *p38α* cells.

Figure S4. Normal adult hippocampal expression of NeuN in *p38α*$^{\Delta Neu}$ mice.

Mean fluorescence intensity of NeuN-positive cells in HC of *p38α*$^{lox/lox}$ and *p38α*$^{\Delta Neu}$ mice. (n = 5) values are mean ± S.E.M. (Student's t-test) ns, non-significant

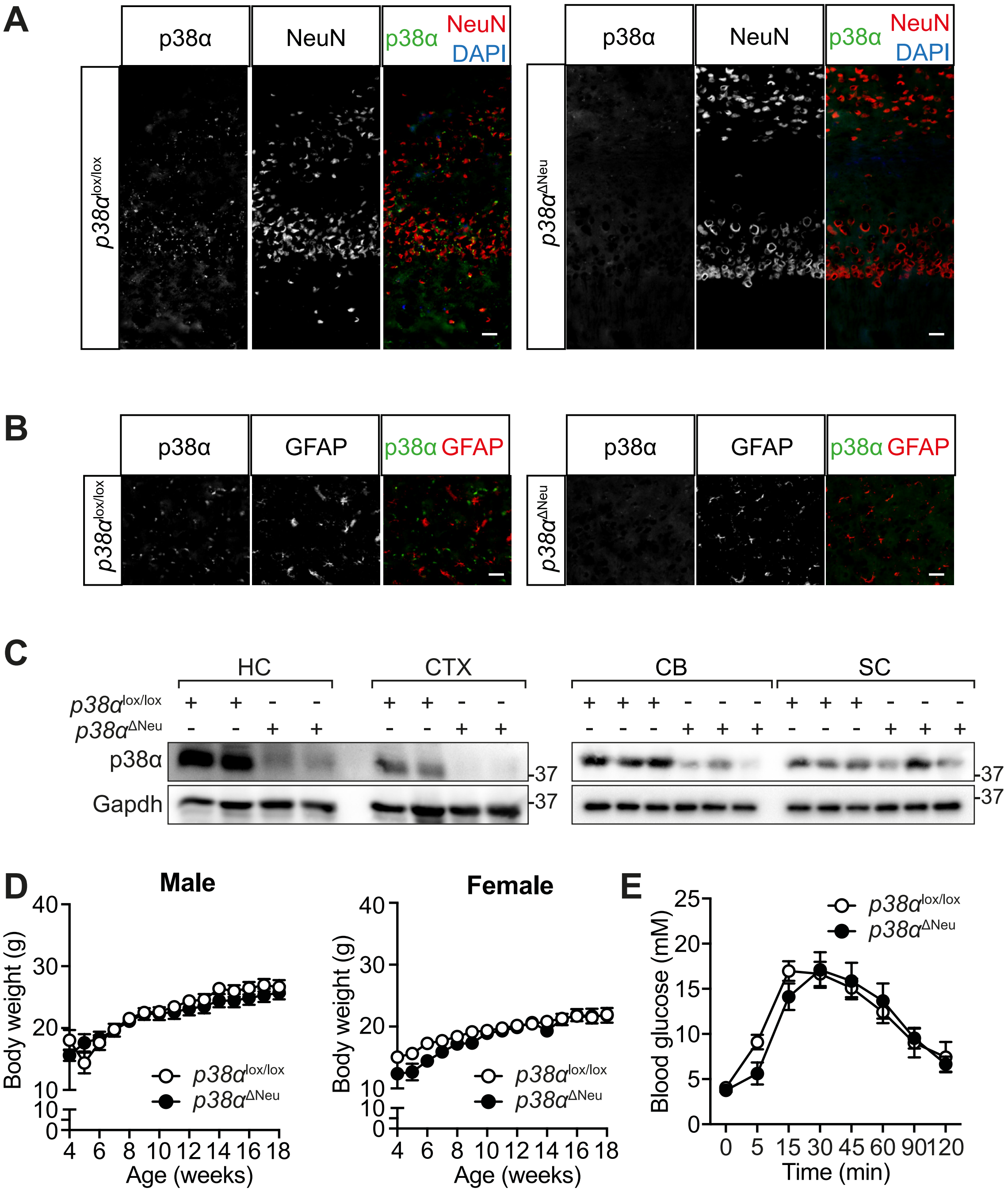
A
p38α
NeuN
p38α NeuN DAPI
p38αlox/lox
p38αΔNeu
B
GFAP
p38α GFAP
C
HC
CTX
CB
SC
p38αlox/lox
p38αΔNeu
p38α
Gapdh
-37
D
Male
Female
Body weight (g)
Age (weeks)
E
Blood glucose (mM)
Time (min)

Figure S1

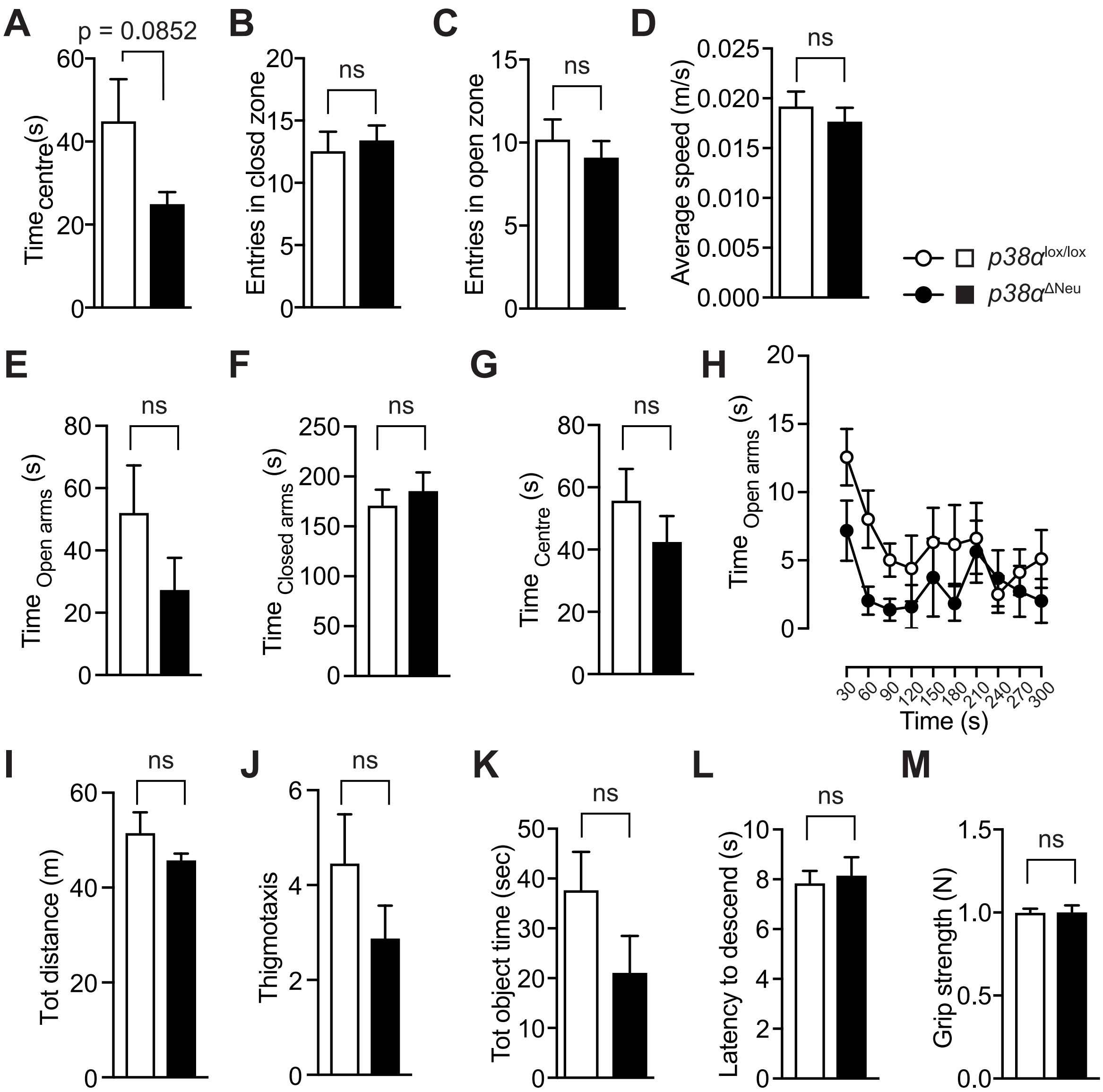
A
p = 0.0852
Time centre (s)
B
ns
Entries in closd zone
C
ns
Entries in open zone
D
ns
Average speed (m/s)
p38α lox/lox
p38α ΔNeu
E
ns
Time Open arms (s)
F
ns
Time Closed arms (s)
G
ns
Time Centre (s)
H
Time Open arms (s)
Time (s)
I
ns
Tot distance (m)
J
ns
Thigmotaxis
K
ns
Tot object time (sec)
L
ns
Latency to descend (s)
M
ns
Grip strength (N)

Figure S2

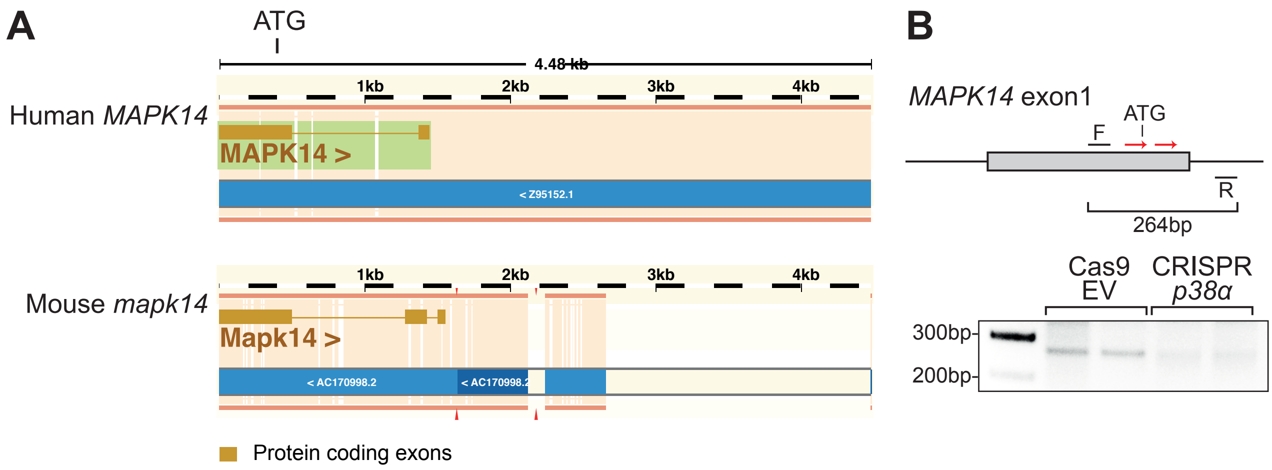
A
ATG
4.48 kb
1kb
2kb
3kb
4kb
Human MAPK14
MAPK14 >
< Z95152.1
Mouse mapk14
Mapk14 >
< AC170998.2
< AC170998.2
Protein coding exons
B
MAPK14 exon1
ATG
F
R
264bp
Cas9
EV
CRISPR
p38α
300bp
200bp

Figure S3

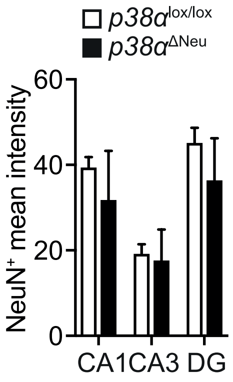
p38α lox/lox
p38α ΔNeu
NeuN+ mean intensity
60
40
20
0
CA1
CA3
DG

Figure S4

Full-length blot images used in Figures 2-4

Fig. 2A

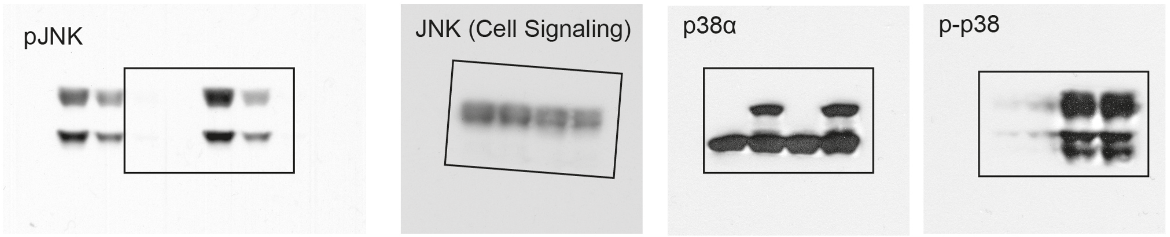

Fig. 2C

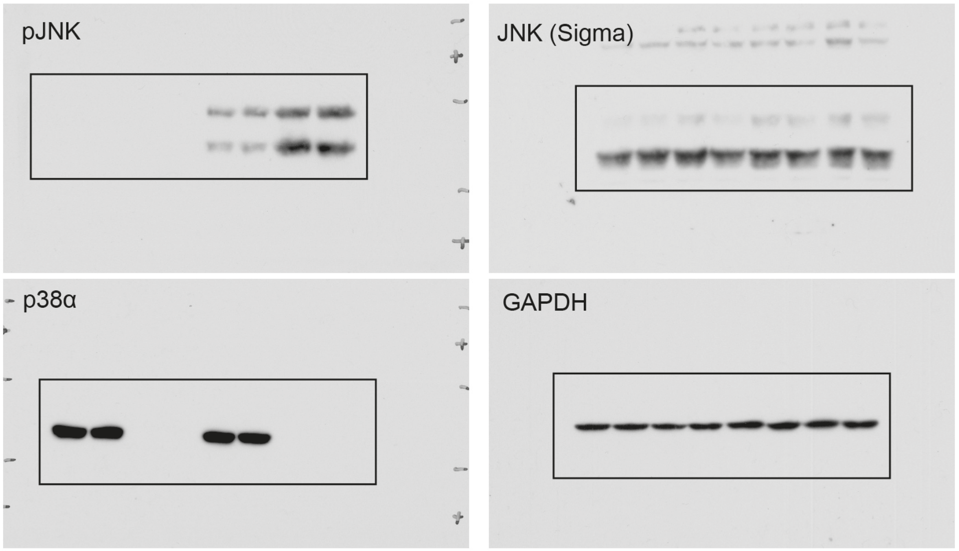

Fig. 2E

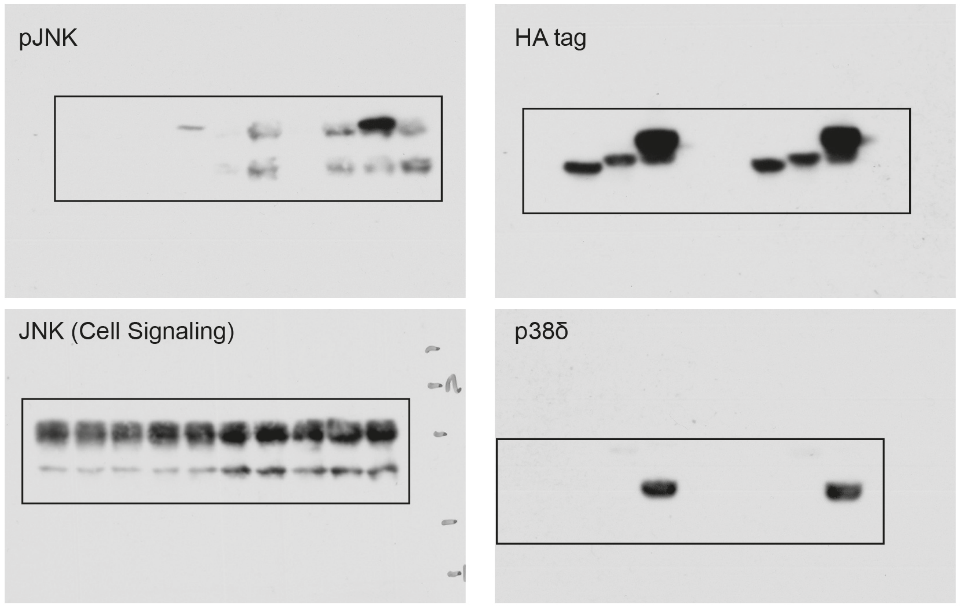

Fig. 3A

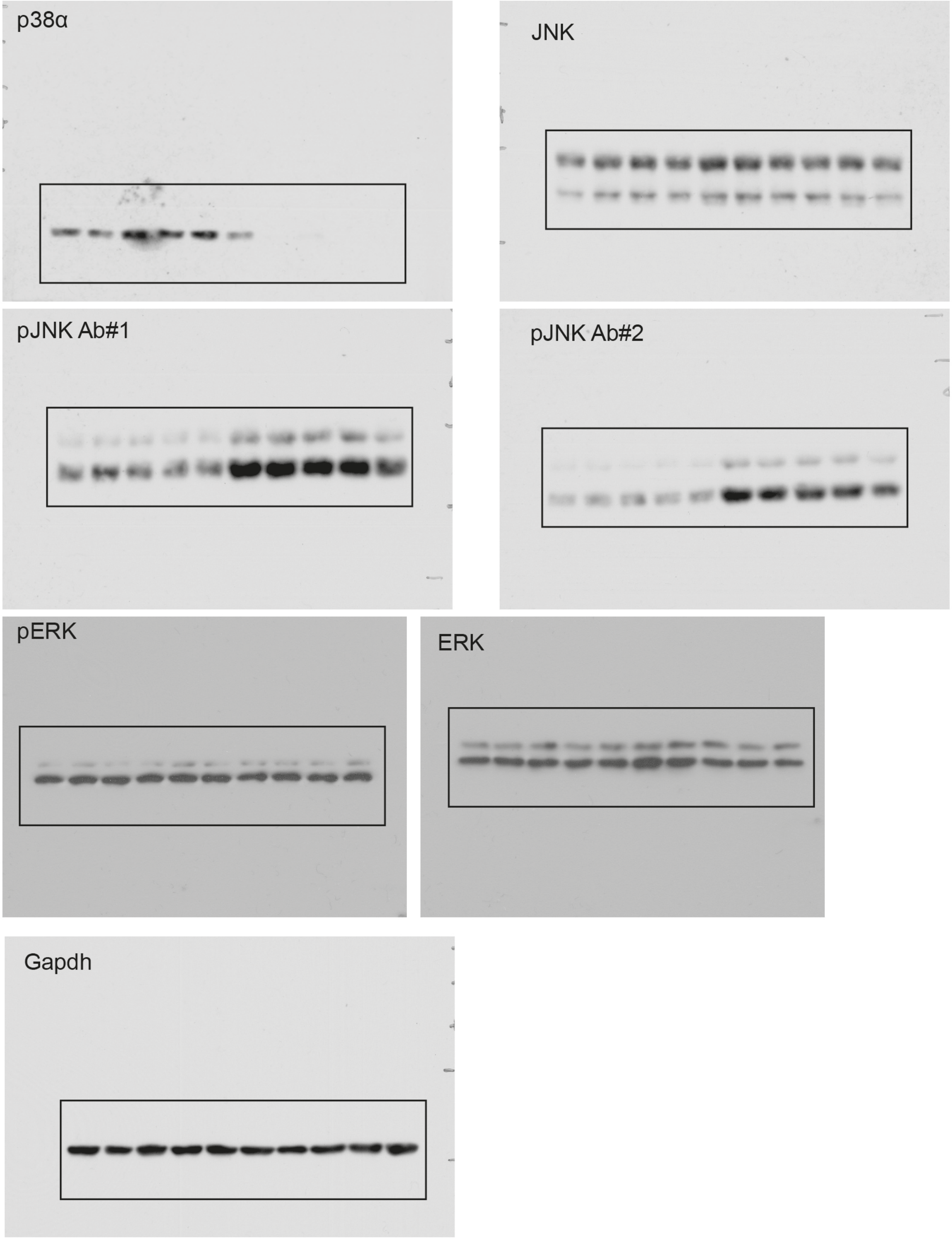

Fig. 4A

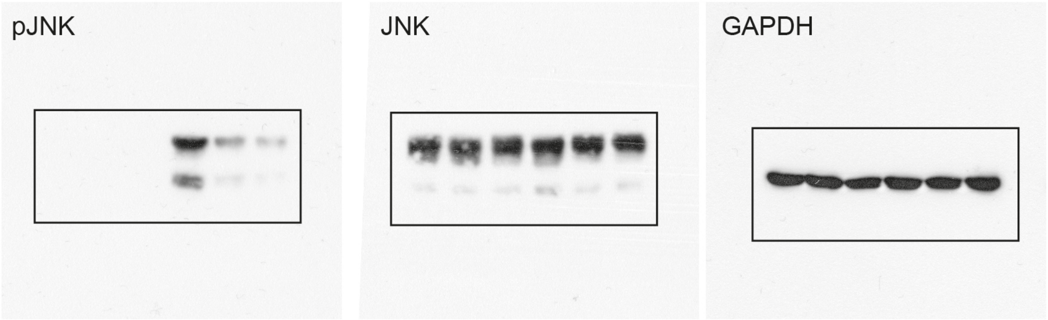

Fig. 4C

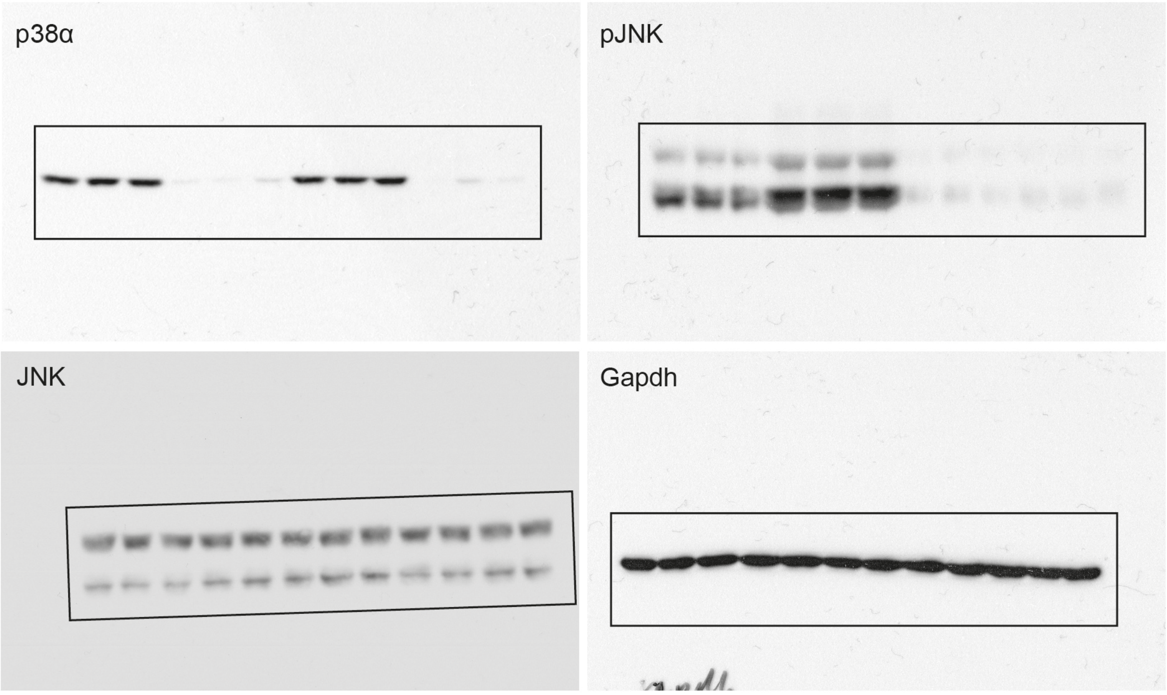

Supplement: Supplementary file 1 — Supplementary information [file 41598_2018_32592_MOESM1_ESM.pdf]
